# Functional comparison of PBMCs isolated by Cell Preparation Tubes (CPT) vs. Lymphoprep Tubes

**DOI:** 10.1186/s12865-020-00345-0

**Published:** 2020-03-30

**Authors:** Han Chen, Christian M. Schürch, Kevin Noble, Kenneth Kim, Peter O. Krutzik, Erika O’Donnell, Jason Vander Tuig, Garry P. Nolan, David R. McIlwain

**Affiliations:** 1 Department of Microbiology & Immunology, Stanford University School of Medicine, Stanford, CA USA; 2 WCCT Global Inc., Cypress, CA USA; 3 ARK Clinical Research, Long Beach, CA USA; 4 Primity Bio Inc., Fremont, CA USA

**Keywords:** Peripheral blood mononuclear cells, BD Cell Preparation Tubes, Lymphoprep Tubes, Flow cytometry, T cell, Cytokine

## Abstract

**Background:**

Cryopreserved human peripheral blood mononuclear cells (PBMCs) are a commonly used sample type for a variety of immunological assays. Many factors can affect the quality of PBMCs, and careful consideration and validation of an appropriate PBMC isolation and cryopreservation method is important for well-designed clinical studies. A major point of divergence in PBMC isolation protocols is the collection of blood, either directly into vacutainers pre-filled with density gradient medium or the use of conical tubes containing a porous barrier to separate the density gradient medium from blood. To address potential differences in sample outcome, we isolated, cryopreserved, and compared PBMCs using parallel protocols differing only in the use of one of two common tube types for isolation.

**Methods:**

Whole blood was processed in parallel using both Cell Preparation Tubes™ (CPT, BD Biosciences) and Lymphoprep™ Tubes (Axis-Shield) and assessed for yield and viability prior to cryopreservation. After thawing, samples were further examined by flow cytometry for cell yield, cell viability, frequency of 10 cell subsets, and capacity for stimulation-dependent CD4+ and CD8+ T cell intracellular cytokine production.

**Results:**

No significant differences in cell recovery, viability, frequency of immune cell subsets, or T cell functionality between PBMC samples isolated using CPT or Lymphoprep tubes were identified.

**Conclusion:**

CPT and Lymphoprep tubes are effective and comparable methods for PBMC isolation for immunological studies.

## Background

Profiling of immune cells with single-cell technologies is a key component of both basic science research, evaluation of therapeutics in clinical trials, and clinical care. Isolation of peripheral blood mononuclear cells (PBMCs) is a mainstay of sample preparation for many single-cell technology applications [1]. PBMCs in particular are a coveted sample type because they are suitable for stable long-term cryopreservation as viable cells facilitating functional analysis up to years after collection [1, 2].

Differences in PBMC sample processing techniques, including cryopreservation and thawing, can have a major influence on yield, viability, and in outcomes of downstream assays [3–5]. Despite the near ubiquity of PBMC isolation, there is little standardization of processing methods across organizations and laboratories, and few studies have directly compared common differences in protocol steps [3–5].

The central principle of PBMC isolation protocols is centrifugal separation of blood components against a high-density medium such as Ficoll-Paque®, Histopaque®-1077, etc. However, use of high-density medium alone requires careful pipetting and handling to prevent inadvertent mixing of layers, and therefore can result in wide range of results between operators and processing runs. To make the implementation of density gradient mediums easier and to achieve greater consistency in PBMC isolation, two primary options have been developed to prevent inadvertent mixing of layers. The first option is to draw blood directly into a vacutainer containing Ficoll-Paque® density gradient medium separated from blood by a thick layer of polyester resin such as in Cell Preparation Tubes (CPT) manufactured by BD Biosciences. The second option is to load diluted blood into tubes containing a high-density gradient medium separated by a plastic barrier (e.g. frit). Plastic barrier-based products include tubes that come pre-filled with high-density separation media, such as Lymphoprep Tubes (Axis-Shield) and EZ Lympho-Sep™ Lymphocyte Separation Tubes (Biological Industries), or those that contain only the polyethylene insert, such as SepMate™ Tubes (STEMCELL Technologies) and Accuspin™ Tubes (Sigma-Aldrich).

Plastic barrier-based tubes are used secondary to phlebotomy and dilution of blood in phosphate buffered saline (PBS) and can accommodate a wide range of input blood volumes, from 0.5 mL to 25 mL, depending on tube size and dilution. CPTs are more restrictive, being available only in 4 mL or 8 mL blood draw sizes. However, because CPTs are used directly for phlebotomy, blood can be centrifuged immediately post blood draw without additional pipetting or dilution steps. Thus, CPTs can be an advantage in busy clinical environments where staff time is limited or when biological safety cabinets are unavailable for the initial processing steps.

To evaluate two frequently used options for simplifying PBMC isolation, we directly compared the performance of PBMC isolation and cryopreservation using CPT versus a plastic barrier-based tube option, Lymphoprep (LP). We assessed the yield, viability, frequencies of 10 unique immune subsets, and cytokine production in both CD4+ (helper) and CD8+ (cytotoxic) T cells in response to two stimulation conditions; each measurement showing no significant differences between the CPT and LP tube methods. These results indicate that CPT and LP tubes are equally suitable for collection of high-quality PBMCs for downstream immunological assays.

## Results

### PBMC recovery and viability are comparable between CPT and Lymphoprep methods

**Fig. 1:**
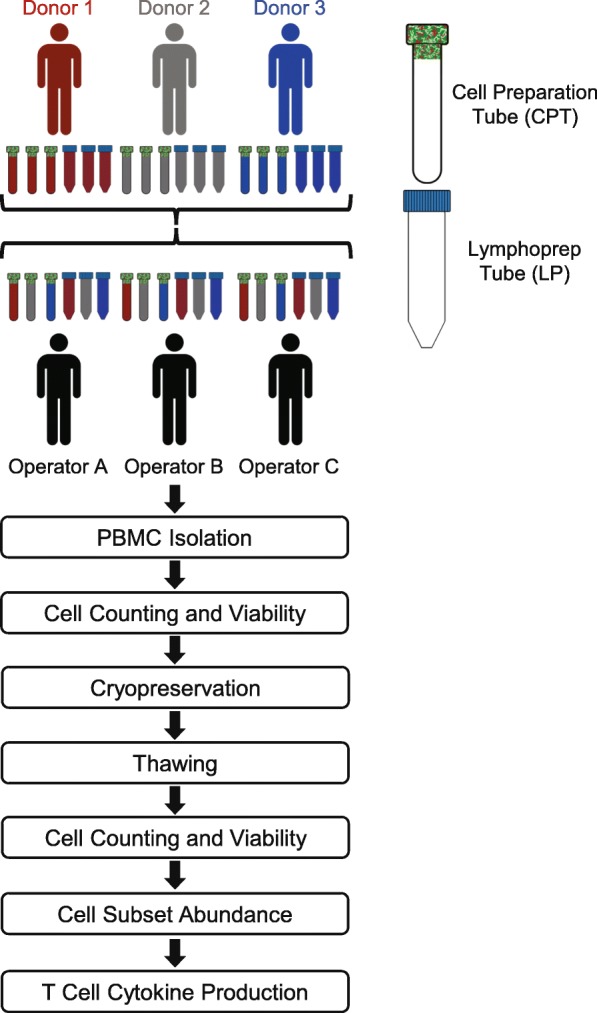
Schematic of the PBMC sample collection and processing. Six aliquots of blood (three for CPT processing and three for LP tube processing) were drawn from three donors. A CPT and LP tube from each donor was processed in parallel by three unique operators. Performance of PBMC isolation methods were measured by their cell yields, viabilities, and recovery both fresh and after cryopreservation and thawing. PBMCs were also stained with surface and intracellular antibodies to assess abundance of cell subsets and functional T cell response following stimulation

Two PBMC processing and cryopreservation methods were designed differing only in use of CPT or LP tubes (see Methods). To examine variability between method, donor, and operator, six peripheral blood samples were obtained from each of three healthy donors at a single timepoint. Three CPT and three standard vacutainers were drawn from each donor. All samples from each donor were processed into PBMCs by three different operators using CPT and LP methods in parallel (Fig. 1).

**Fig. 2:**
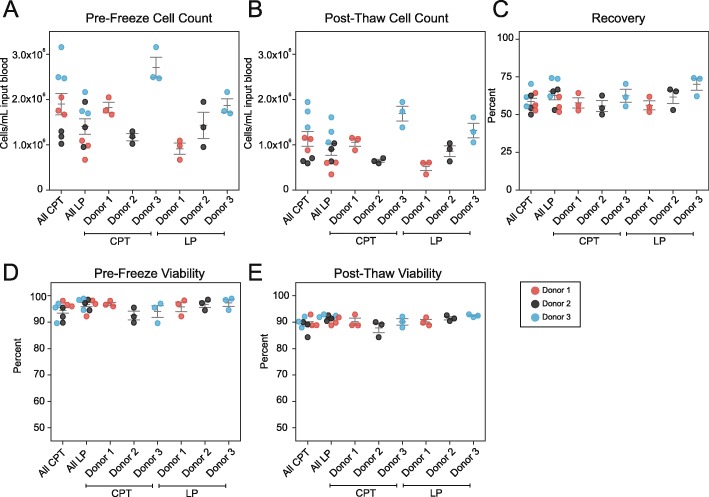
Equivalent PBMC yield and post-thaw recovery using CPT or LP Tubes. **a-e** Three healthy donor blood draws of six tubes each were split between three operators for parallel PBMC isolation using CPT and LP Tube methods. Total PBMCs were counted and assessed for viability by acridine iodine and propidium iodide staining using a Cellometer K2 immediately after isolation (pre-freeze), and by near-IR viability staining using a BD LSRII flow cytometer after cryopreservation and recovery (post-thaw). **a** The yield of PBMCs per mL of input blood pre-freeze. **b** The yield of PBMCs per mL of input blood post-thaw. **c** Percent of cell recovery post-thaw. **d** Cell viability pre-freeze. **e** Cell viability post-thaw. Horizontal lines indicate mean +/− SEM. Donors 1,2, 3 are depicted by red, black, and blue dots respectively. Three dots are shown per donor which represent single samples processed by three different operators. For statistical analysis for all figures see Table 1

To determine recovery rates, yield and viability of PBMCs isolated by both CPT and LP methods were determined immediately after PBMC isolation (fresh), as well as after cryopreservation for 1 month in liquid nitrogen. CPT and LP methods resulted in an average of yield of 1.9 × 10^6^ (± 0.2 × 10^6^) and 1.4 × 10^6^ (± 0.2 × 10^6^) cells/mL input blood respectively prior to freezing **(**Fig. 2**)**. After thawing CPT and LP methods yielded 1.1 × 10^6^ (± 0.2 × 10^6^) and 0.9 × 10^6^ (± 0.1 × 10^6^) cells, respectively. Similar results were obtained for an additional set of samples examined before cryopreservation and after thawing using a hematology analyzer **(**Additional File 1: Figure S1, Additional File 2: Table S1). Viability was high for both methods with average values of 94.5 and 96.6% pre-freeze and 89.4 and 91.4% post-thaw for CPT and LP respectively. Overall yield, post-thaw recovery, and viability were within expected ranges and not significantly different between CPT and LP methods [all *p*-values greater than 0.05, Wilcoxon] (Table 1).

**Table 1:** Statistical analysis shows choice of CPT or LP method is not the major contributor to variance for all parameters measured. Anova variance for contribution of donor, method (CPT or LP), and operator on yield and viability, cell population distribution (populations), and stimulated cytokine expression in CD4+ and CD8+ T cells (CEF stimulated, PMA-I stimulated). Sum squares, sum square contributions, *F* values, and *P* values are show for each parameter measured (rows)

**Yield and Viability**						**Populations**						**Populations**					
	**Wilcoxon*P*Value**	**Sum Square**	**Sum Square Contribution**	**ANOVA*F*Value**	**ANOVA*P*Value**		**Wilcoxon*P*Value**	**Sum Square**	**Sum Square Contribution**	**ANOVA*F*Value**	**ANOVA*P*Value**		**Wilcoxon*P*Value**	**Sum Square**	**Sum Square Contribution**	**ANOVA*F*Value**	**ANOVA*P*Value**
**Cells/mL Pre Freeze**						**Monocytes**						**Memory CD4+**					
*Method*	9.77E-02	1.11E+ 12	21.4%	6.49	2.56E-02	*Method*	5.73E-01	9.34E-01	0.4%	0.34	5.70E-01	*Method*	3.01E-01	8.93E-02	0.0%	0.04	8.45E-01
*Donor*		3.65E+ 12	70.8%	10.73	2.13E-03	*Donor*		2.43E+ 02	94.0%	44.57	2.79E-06	*Donor*		9.39E+ 02	98.3%	209.59	4.65E-10
*Operator*		4.01E+ 11	7.8%	1.18	3.41E-01	*Operator*		1.45E+ 01	5.6%	2.66	1.10E-01	*Operator*		1.65E+ 01	1.7%	3.68	5.66E-02
**Cells/mL Post Freeze**						**Lymphocytes**						**Naïve CD4+**					
*Method*	1.29E-01	2.42E+ 11	9.7%	3.39	9.06E-02	*Method*	4.26E-01	1.60E+ 00	0.4%	0.60	4.53E-01	*Method*	4.26E-01	1.02E+ 00	0.1%	0.61	4.50E-01
*Donor*		2.15E+ 12	86.3%	15.07	5.33E-04	*Donor*		3.40E+ 02	95.0%	63.76	4.05E-07	*Donor*		1.19E+ 03	99.3%	355.42	2.09E-11
*Operator*		1.01E+ 11	4.0%	0.70	5.14E-01	*Operator*		1.61E+ 01	4.5%	3.03	8.62E-02	*Operator*		6.94E+ 00	0.6%	2.07	1.68E-01
**% Recovery**						**NK Cells**						**Memory CD4-**					
*Method*	1.29E-01	7.26E+ 01	12.0%	2.47	1.42E-01	*Method*	8.23E-01	8.48E-01	0.5%	0.31	5.91E-01	*Method*	2.50E-01	1.61E+ 00	0.6%	1.39	2.62E-01
*Donor*		2.93E+ 02	48.4%	4.97	2.68E-02	*Donor*		1.69E+ 02	95.5%	30.39	2.01E-05	*Donor*		2.74E+ 02	98.2%	118.24	1.27E-08
*Operator*		2.40E+ 02	39.6%	4.07	4.48E-02	*Operator*		7.15E+ 00	4.0%	1.29	3.12E-01	*Operator*		3.42E+ 00	1.2%	1.47	2.68E-01
**Viability Pre Freeze**						**T Cells**						**Naïve CD4-**					
*Method*	1.64E-01	2.05E+ 01	26.6%	3.72	7.79E-02	*Method*	9.77E-02	9.44E+ 00	5.8%	3.08	1.05E-01	*Method*	2.50E-01	3.40E+ 00	2.0%	2.35	1.51E-01
*Donor*		9.17E+ 00	11.9%	0.83	4.59E-01	*Donor*		1.22E+ 02	74.5%	19.87	1.55E-04	*Donor*		1.51E+ 02	90.4%	52.13	1.21E-06
*Operator*		4.73E+ 01	61.5%	4.29	3.92E-02	*Operator*		3.22E+ 01	19.7%	5.26	2.29E-02	*Operator*		1.25E+ 01	7.5%	4.34	3.83E-02
**Viability Post Freeze**						**B Cells**											
*Method*	9.77E-02	1.79E+ 01	58.7%	4.10	6.56E-02	*Method*	3.59E-01	1.23E-01	0.9%	0.27	6.12E-01						
*Donor*		8.18E+ 00	26.8%	0.94	4.18E-01	*Donor*		1.24E+ 01	92.7%	13.76	7.84E-04						
*Operator*		4.41E+ 00	14.5%	0.51	6.15E-01	*Operator*		8.63E-01	6.4%	0.95	4.13E-01						
**CEF Stimulated**						**PMA-I Stimulated**						**Unstimulated**					
	**Wilcoxon*P*Value**	**Sum Square**	**Sum Square Contribution**	**ANOVA*F*Value**	**ANOVA*P*Value**		**Wilcoxon*P*Value**	**Sum Square**	**Sum Square Contribution**	**ANOVA*F*Value**	**ANOVA*P*Value**		**Wilcoxon*P*Value**	**Sum Square**	**Sum Square Contribution**	**ANOVA*F*Value**	**ANOVA*P*Value**
**CD4 + IFNg+**						**CD4 + IFNg+**						**CD4 + IFNg+**					
*Method*	8.20E-01	2.80E-06	0.4%	0.19	6.74E-01	*Method*	4.96E-01	7.70E-01	0.7%	0.52	4.87E-01	*Method*	9.10E-01	2.94E-07	0.0%	0.03	8.69E-01
*Donor*		7.74E-04	97.9%	25.65	4.64E-05	*Donor*		1.08E+ 02	99.0%	36.09	8.39E-06	*Donor*		6.38E-04	99.1%	30.97	1.83E-05
*Operator*		1.40E-05	1.8%	0.46	6.40E-01	*Operator*		3.10E-01	0.3%	0.10	9.02E-01	*Operator*		5.31E-06	0.8%	0.26	7.77E-01
**CD4 + IL-17+**						**CD4 + IL-17+**						**CD4 + IL-17+**					
*Method*	6.73E-01	5.00E-09	0.1%	0.01	9.10E-01	*Method*	4.96E-01	2.35E-02	2.1%	0.38	5.48E-01	*Method*	5.79E-02	4.36E-07	27.8%	3.20	9.87E-02
*Donor*		3.30E-06	81.2%	4.43	3.63E-02	*Donor*		1.03E+ 00	92.8%	8.36	5.31E-03	*Donor*		7.60E-07	48.4%	2.80	1.01E-01
*Operator*		7.60E-07	18.7%	1.02	3.90E-01	*Operator*		5.57E-02	5.0%	0.45	6.46E-01	*Operator*		3.73E-07	23.8%	1.37	2.90E-01
**CD4 + IL-4+**						**CD4 + IL-4+**						**CD4 + IL-4+**					
*Method*	1.64E-01	6.47E-04	53.1%	1.71	2.16E-01	*Method*	2.50E-01	4.74E-01	8.3%	1.83	2.01E-01	*Method*	4.26E-01	2.55E-04	8.8%	0.44	5.18E-01
*Donor*		3.53E-04	28.9%	0.47	6.38E-01	*Donor*		4.92E+ 00	86.3%	9.50	3.36E-03	*Donor*		1.38E-03	47.7%	1.21	3.33E-01
*Operator*		2.20E-04	18.0%	0.29	7.53E-01	*Operator*		3.08E-01	5.4%	0.59	5.68E-01	*Operator*		1.27E-03	43.6%	1.10	3.63E-01
**CD8 + IFNg+**						**CD8 + IFNg+**						**CD4-IFNg+**					
*Method*	3.74E-01	1.49E-03	0.2%	0.11	7.45E-01	*Method*	3.59E-01	1.61E+ 01	0.4%	1.01	3.35E-01	*Method*	6.78E-01	1.20E-05	9.5%	0.78	3.93E-01
*Donor*		9.01E-01	91.6%	33.38	1.25E-05	*Donor*		4.09E+ 03	99.3%	128.58	7.85E-09	*Donor*		8.00E-06	6.3%	0.26	7.74E-01
*Operator*		8.09E-02	8.2%	3.00	8.80E-02	*Operator*		1.39E+ 01	0.3%	0.44	6.55E-01	*Operator*		1.07E-04	84.2%	3.49	6.38E-02
**CD8 + IL-17+**						**CD8 + IL-17+**						**CD4-IL-17+**					
*Method*	1.98E-01	6.81E-07	20.2%	3.65	8.04E-02	*Method*	3.01E-01	1.77E-03	3.8%	1.07	3.20E-01	*Method*	4.19E-01	1.28E-06	17.4%	1.77	2.08E-01
*Donor*		2.43E-06	71.9%	6.52	1.21E-02	*Donor*		4.06E-02	85.9%	12.28	1.25E-03	*Donor*		5.61E-06	76.2%	3.88	5.03E-02
*Operator*		2.68E-07	7.9%	0.72	5.08E-01	*Operator*		4.91E-03	10.4%	1.49	2.65E-01	*Operator*		4.74E-07	6.4%	0.33	7.27E-01
**CD8 + IL-4+**						**CD8 + IL-4+**						**CD4-IL-4+**					
*Method*	7.34E-01	1.02E-04	9.9%	0.31	5.87E-01	*Method*	3.59E-01	6.41E-03	0.0%	0.07	7.92E-01	*Method*	1.00E+ 00	1.72E-05	0.4%	0.02	8.85E-01
*Donor*		4.89E-04	47.5%	0.74	4.96E-01	*Donor*		1.62E+ 01	98.6%	91.81	5.33E-08	*Donor*		2.95E-03	73.3%	1.86	1.99E-01
*Operator*		4.37E-04	42.5%	0.67	5.32E-01	*Operator*		2.16E-01	1.3%	1.23	3.27E-01	*Operator*		1.05E-03	26.2%	0.66	5.33E-01

### Distribution of immune cell subsets in isolated PBMCs is unaffected by CPT or Lymphoprep methods

**Fig. 3:**
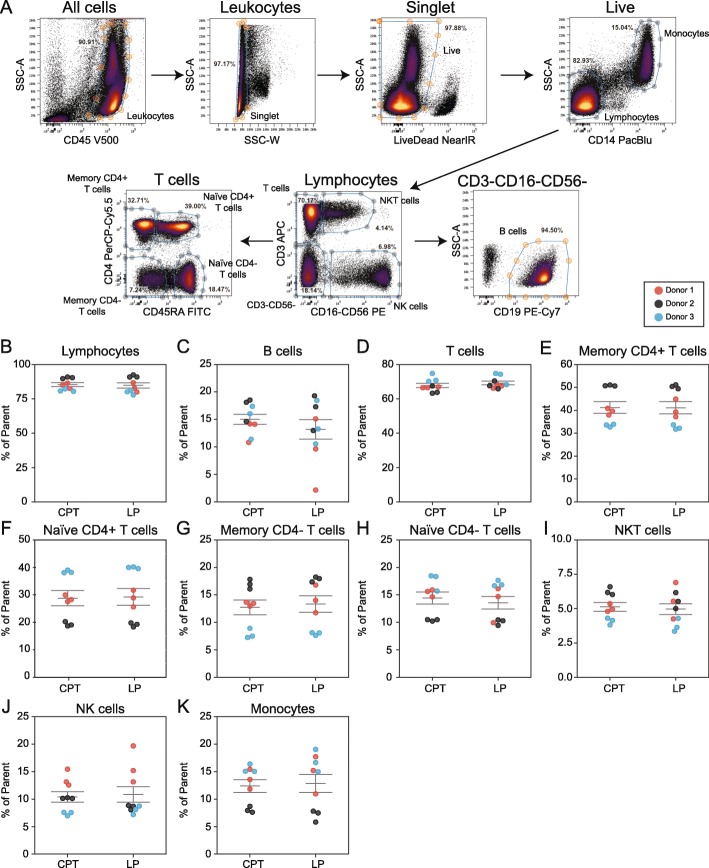
Similar distribution of immune cell subsets in PBMCs isolated with CPT or LP Tubes. **a** Gates used to define ten major indicated cell populations in flow cytometry data from one representative PBMC sample (donor 3). **b-k** Frequencies of cell populations (% of parent gate) in PBMCs isolated by CPT or LP methods determined by flow cytometry for **b** Lymphocytes, **c** B cells, **d** T cells, **e** CD4+ memory T cells, **f** CD4+ naïve T cells, **g** CD4- Naïve T cells, **h** CD4- memory T cells, **i** NKT cells, **j** NK cells, and **k** monocytes. Note: Lymphocytes used as parent gate for B cells statistics. Horizontal lines indicate mean +/− SEM. Donors 1,2, 3 are depicted by red, black, and blue dots respectively. Three dots are shown per donor which represent single samples processed by three different operators

The potential bias of different PBMC processing methods for inadvertently depleting certain cell subsets (e.g. B cells) is a frequent point of concern when designing clinical studies. Therefore, flow cytometry was used to assess frequencies of 10 immune cell populations in PBMC samples isolated using CPT or LP methods. Antibodies recognizing CD14, CD16, CD56, CD19, CD3, CD4, and CD45RA were used to identify total lymphocytes, B cells, T cells, CD4+ memory T cells, CD4+ naïve T cells, CD4- memory T cells, CD4- naïve T cells, NKT cells, NK cells, and monocytes (Fig. 3a). Data for Donor 3 is shown as an example. Frequencies of each population were not significantly different between the two isolation methods indicating no systematic loss of any cell type between CPT versus LP tubes (Fig. 3b-k**,** Table 1).

### CPT and Lymphoprep isolation methods result in similar T cell cytokine functionality

**Fig. 4:**
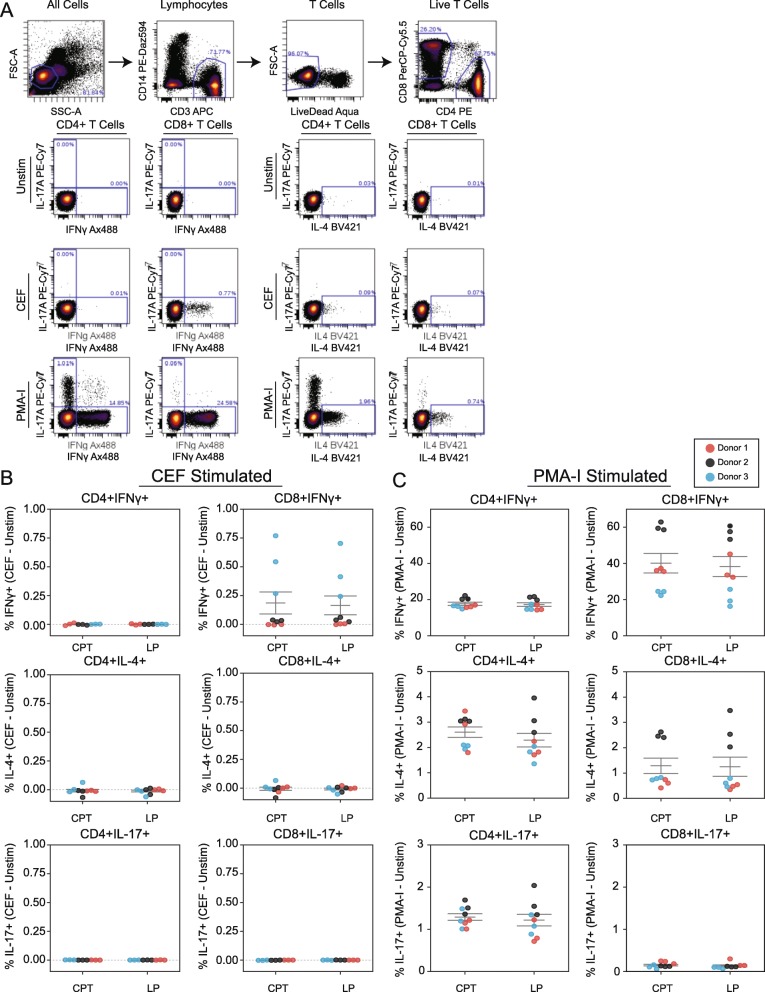
CPT and LP methods maintain equivalent T cell functionality in isolated PBMCs. **a** Gating to identify CD4+ and CD8+ T cells (upper) and biaxial plots showing intracellular cytokine production for IL-17A, IFNγ, and IL-4 in CD4+ and CD8+ T cells after stimulation with CEF, PMA-I, or unstimulated control (unstim). One representative PBMC sample is shown. **b, c** Quantification of indicated intracellular cytokine production in CD4+ and CD8+ T cells from PBMCs isolated by CPT or LP methods following stimulation with **b** CEF or **c** PMA-I. All data generated by flow cytometry; values shown are normalized to unstimulated control (value subtracted by unstimulated control), horizontal lines indicate mean +/− SEM. Donors 1,2, 3 are depicted by red, black, and blue dots respectively. Three dots are shown per donor which represent single samples processed by three different operators

To determine whether T cell functionality is differentially affected by CPT or LP methods, PBMCs were used to assay CD4+ and CD8+ T Cell subsets’ capability to express IFNγ, IL-4, and IL-17 in response to three conditions: 1) unstimulated control, 2) stimulation using a peptide pool containing proteins from cytomegalovirus, Epstein-Barr virus, and influenza virus (CEF peptide pool), and 3) stimulation with phorbol-myristate-acetate and ionomycin (PMA-I) (Fig. 4a).

As expected, relative to unstimulated controls, CEF stimulation resulted in a significant increase in CD8 + IFNγ+ T cells and PMA-I stimulation resulted in significant increases of IFNγ+, IL-4, and IL-17 in both CD4+ and CD8+ T cell populations **(**Fig. 4b, c). For each stimulation condition, there were no significant differences in the ratio of IFNγ, IL-4, and IL-17 positive T cells between the CPT or LP methods, indicating that both preserve a similar degree of T cell functionality after cryopreservation and recovery (Table 1).

### Observed variation is driven primarily by donor-to-donor variability

A three-way ANOVA test was performed to identify what impact the donor, operator, and method had on the yield, viability, and recovery of PBMCs as well as the distribution of immune subsets and the cytokine production of T cell subsets. Overall, donor-to-donor differences accounted for the largest variance in the measured parameters (an average contribution of 74.5 ± 27.5%), compared to the variance contributions from operators (16.1 ± 20.1%) or PBMC isolation method (9.4 ± 14.5%) (Table 1).

## Discussion

The performance of CPT and LP tube methods to isolate PBMCs from peripheral blood was evaluated for cell recovery, viability, frequency of recovered cell subsets, and functional assessment of T cell cytokine production. No significant differences were found between CPT or LP tube methods. Parallel processing of blood from three donors by three operators allowed for the examination of the contribution of method, donor, or operator to observed results. Donor, but not method nor operator, was the major driver of variance in collected data, suggesting that that donor-dependent differences in underlying physiology rather than technical differences were the largest contributors to variation in our observations [6].

Cryopreservation and thawing resulted in considerable cell loss, likely due to a combination of stress from freezing and cell loss associated with additional washing and aspiration steps. These effects are well-known, and our post-thaw recovery was in line with prior reports [3]. No major differences in post-thaw recovery were attributed to the use of CPT versus LP tubes.

The frequency of identifiable immune cell subsets in collected PBMCs were within expected ranges [7, 8]. Not surprisingly, cell subset frequencies varied between donors, but the proportion of populations remained similar within donors regardless of processing by CPT or LP tube with no significant differences between methods. Although intra-donor variability in cell type frequency was generally low, the largest intra-donor variability was observed for B cells in PBMCs processed with LP tubes; consistent with the notion that this cell type is particularly sensitive to the stresses of cryopreservation and thawing [9].

Examination of stimulation-dependent intracellular cytokine production in CD4+ and CD8+ T cells is a key method used to gauge immunological memory in many studies. There were no significant differences in the capacity of T cells from PBMC samples isolated by either CPT or LP methods to activate cytokine production following CEF peptide pool or PMA-I stimulation. As in previous studies, these responses were largely donor dependent [6]. As expected, the CEF peptide pool resulted in IFNγ production in CD8+ T cells [10], while PMA-I caused a release of IFNγ, IL-4, and IL-17 in both CD4+ and CD8+ T cells [11, 12]. The ability of T cells to effectively respond to a specific stimulus (a CEF peptide pool) and a broad and non-specific stimulus (PMA-I) indicates that both methods are well suited for generating PBMC samples functional for T cell studies.

Prior reports have found comparable cellular yield, viability, and functional CD8+ T cell IFNγ responses of PBMCs isolated with CPTs when compared to standard Ficoll-Paque techniques [13, 14]. Another study found the overall performance of PBMCs isolated with CPTs and SepMate Tubes (comparable to LP tubes) to be similar, but superior than those isolated using Ficoll-Paque alone [15]. For example, in that study, cells isolated with either CPT or SepMate resulted in a greater number of PBMCs and higher IFNγ expression after staphylococcal enterotoxin B (SEB) stimulation than PBMCs isolated using only Ficoll-Paque gradient [15].

As described here, two frequently used options for simplifying PBMC isolation, CPT and LP tubes, performed similarly well when tested here in a direct comparison. Given that both tubes resulted in similar performance, researchers might instead focus on other strengths and weaknesses of each tube when making decisions for clinical studies. For instance, CPT tubes can greatly simplify PBMC processing by eliminating a dilution and overlay step prior to centrifugation and allow greater flexibility for shipping centrifuged vacutainers for delayed off-site processing. Plastic barrier-based tubes such as LP on the other hand, can accommodate larger blood volumes which can streamline the processing of large quantities of PBMCs from single blood draws. In addition to these technical considerations, differences in laboratory infrastructure can also dictate feasibility of different PBMC isolation methods. Plastic-barrier based tubes are sold in standard plastic tube sizes (12 mL round bottom, 15 mL conical, 50 mL conical) which are easily accommodated in standard benchtop swinging bucket centrifuges. Glass CPT tubes, in particular the 8 mL blood draw sized CPTs (measured at 16 mm × 125 mm, excluding stopper), do not fit in many standard centrifuge buckets, especially under their aerosol caps. The long length of 8 mL CPT tubes and their glass construction increase the potential for tube breakage if improper loading results in contact with rotors, or if the tubes are centrifuged above recommended speeds. Finally, glass tubes in general are incompatible with biosafety regulations in some laboratory settings.

## Conclusions

To our knowledge, this is the first study that directly compares the performance of CPT and LP tubes. The results suggest that both CPT and LP Tubes are both effective methods of PBMC isolation that result in similar cell yield, viability, frequency of subsets, and capacity for stimulation-dependent T cell intracellular cytokine production.

## Materials and methods

### Blood collection

Peripheral blood samples from three adult volunteers were obtained at a single time point using three 8 mL Cell Preparation Tubes (CPT) with sodium heparin (BD Biosciences) and three 10 mL Vacutainer plastic blood collection tubes with sodium heparin (BD Biosciences). Each sample was processed within 30 min of collection and processed in parallel by three different operators who each isolated PBMCs from each donor using two methods: isolation by CPT tubes and isolation using LP tubes (Axis-Shield) (see below). De-identified blood samples were obtained from healthy adult volunteers following the guidelines of the Environmental Health and Safety Biosafety program of Stanford University. Donors provided informed consent in accordance with IRB protocols approved by the Stanford University Administrative Panel on Human Subjects Research.

### PBMC isolation with CPT

Whole blood was collected into an 8 mL sodium heparinized CPT vacutainer and inverted multiple times to ensure homogenization of the sodium heparin anti-coagulant and blood. The vacutainer was centrifuged at 1700 x g for 20 min at 21 °C, resulting in the separation of contents into layers: the upper layer containing plasma with a cloudy band of PBMCs, the middle layer containing a think polyester resin, and the lower layer containing erythrocytes and granulocytes. After centrifugation, the CPT was gently inverted 10 times to resuspend PBMCs and plasma, then decanted into a 15 mL conical tube pre-filled with 8 mL of Dulbecco’s Phosphate-Buffered Saline (PBS, Thermo Scientific). The capped 15 mL conical tube was mixed by inversion and centrifuged at 300 x g for 15 min at 4 °C. The supernatant was carefully aspirated, the cell pellet was resuspended in 10 mL of fresh PBS, and centrifuged for a subsequent 10 min at 300 x g at 4 °C. Following this centrifugation step, the supernatant was again carefully aspirated without disturbing the cell pellet, and the pelleted PBMCs were resuspended in 3 mL of fetal bovine serum (FBS, HyClone). Five hundred microliter of cells were aliquoted into six 2 mL cryovials, each pre-filled with 500 μL of freezing medium composed of FBS and 20% DMSO (Sigma Hybri-Max D2650) for a final DMSO concentration of 10%. The filled cryovials were placed in CoolCell freezing containers (Biocision) at − 80 °C for 24 h prior to transfer to liquid nitrogen for long-term storage. Residual volume of PBMCs in FBS were retained and used for counting and viability assessment.

### PBMC isolation with Lymphoprep tubes

In preparation for PBMC isolation, Lymphoprep tubes were first centrifuged at 500 x g for 1 min at 21 °C to ensure displacement of the pre-filled Lymphoprep media to the bottom of the tube. Whole blood was collected by phlebotomy into a 10 mL sodium heparin vacutainer and inverted to ensure mixing of blood with anti-coagulant. Nine milliliters of blood were transferred into a 50 mL conical tube pre-filled with 10 mL PBS for a ~ 1:1 dilution. This diluted blood was decanted into the upper chamber of a Lymphoprep tube, separated by a porous polyethylene insert from the lower Ficoll-Paque-containing chamber. The filled Lymphoprep tube was centrifuged at 800 x g for 20 min 21 °C with no brake. Centrifugation resulted in an upper layer of plasma with a cloudy band of PBMCs and a lower layer of erythrocytes and polymorphonuclear cells, separated by the polyethylene insert. After centrifugation, the cloudy PBMC band was collected using a sterile transfer pipette and added to a 15 mL conical tube pre-filled with 10 mL of PBS. The capped 15 mL conical tube was mixed by inversion and centrifuged at 300 x g for 15 min at 4 °C. The supernatant was carefully aspirated, the cell pellet was resuspended in 10 mL of fresh PBS, and centrifuged for a subsequent 10 min at 300 x g at 4 °C. Following this centrifugation step, the supernatant was again carefully aspirated without disturbing the cell pellet, and PBMCs were resuspended in 3 mL of FBS. Five hundred microliter of cells were aliquoted into six 2 mL cryovials each pre-filled with 500 μL of freezing medium composed of FBS and 20% DMSO for a final DMSO concentration of 10%. The filled cryovials were placed in CoolCell freezing containers at − 80 °C for 24 h prior to transfer to liquid nitrogen for long-term storage. Residual volume of PBMCs in FBS were retained and used for counting and viability assessment.

### Pre-freeze cell counting and viability

Residual volumes of PBMCs in FBS from each sample were used to assess the PBMC yield and viability prior to cryopreservation. PBMCs were stained with acridine orange (AO) and propidium iodine (PI) to identify all nucleated cells and dead nucleated cells, respectively. Equal volumes of PBMCs and Cellometer Via Stain AOPI staining Solution (Nexelom Biosciences) were combined for staining. 20μL of stained PBMCs were dispensed into a disposable Cellometer Counting Chamber and placed into a Cellometer K2 instrument (Nexelom Biosciences) for automated counting and viability determination. Where indicated, additional PBMC counts were obtained using a Sysmex XE-2100 automated hematology analyzer (Sysmex Corporation) according to manufacturer’s protocol.

### Thawing of PBMCs

Frozen cryovials of PBMCs were taken from liquid nitrogen storage were placed in a 37 °C water bath until thawed and the contents were transferred to a 15 mL conical tube containing pre-warmed media composed of complete RPMI (cRPMI) (RPMI-1640 [Gibco] containing 10% FBS [HyClone] and Benzonase nuclease [≥ 250 units/μL, Sigma]). Samples were then centrifuged at 300 x g for 5 min at room temperature and resuspended thoroughly in pre-warmed cRPMI and Benzonase. A small aliquot of the cell suspension was aliquoted for counting and viability staining. The remaining cells for antibody staining were centrifuged again at 300 x g for 5 min at room temperature, resuspended in cRPMI, and placed at room temperature until cell counting was complete. Cells were divided into two aliquots, one for surface antibody staining analysis and one for intracellular antibody staining analysis.

### Post-thaw cell counting and viability

Leukocyte viability stain (detectable in near-IR channels) was added into each 100 μL cell suspension aliquot and incubated at 21 °C for 10 min. Three milliliter of FBS stain buffer (BD Biosciences) was added to each cell sample for washing and centrifuged at 300 x g for 5 min at 21 °C. The supernatant was aspirated, and cells were resuspended in a solution containing a final concentration of 1025 beads/μL (123count beads, eBioscience) and 1.6% PFA (Electron Microscopy Sciences). Following a 10-min incubation at 21 °C, viability was assessed by running the samples through a flow cytometer. Where indicated, additional cell counts were obtained using a Sysmex XE-2100 hematology analyzer according to manufacturer’s protocol.

### Surface staining of PBMCs

Cells were washed in 150 μL of BSA Stain Buffer (BD Biosciences) in preparation of phenotype staining. 50μL of Fc block cocktail (composed of Fc Block [Biolegend], Live Dead Near IR Fixable Viability Stain (Thermo Fisher), BSA Stain Buffer) was added to each sample and incubated for 10 min at room temperature. After the incubation period, cells were stained with a monoclonal antibody cocktail containing CD19-PacBlue, CD45-PE, CD45RA-FITC, CD4-PerCP-Cy5.5, CD16-PE, CD56-PE, CD19-PE-Cy7, and CD3-APC. All antibodies were received from Biolegend and diluted at a 1:50 ratio in BSA stain buffer. Following a 30-min staining period at 4 °C, samples were washed by centrifuging at 400 x g for 5 min (room temperature) and resuspended in BSA stain buffer. Following the third centrifugation step, PBMCs were resuspended in BSA Stain Buffer and 16% PFA was added to each sample for a final concentration of 1.6% PFA. After a 10-min fixation period at 21 °C, cells were washed and resuspended in BSA Stain Buffer for flow cytometry acquisition.

### Stimulation of PBMCs for cytokine production

Stimulation of PBMCs using a CEF (cytomegalovirus, Epstein-Barr virus, influenza virus) peptide pool (Anaspec; 1 μg/mL) and a Phorbol 12-myristtate 13-acetate (PMA; Sigma; 50 ng/mL) + Ionomycin (Thermo Fisher; 1 μg/mL) cocktail was performed and compared to an unstimulated control. Following the thawing and counting procedure, cells were centrifuged and resuspended in 350 μL of fresh cRPMI. Cells were then transferred into a 96-well, round-bottom plate and rested at 37 °C for up to 2 h. During this period, a 2X CEF cocktail (4 μL of 5000x CEF and 20 μL of 1000X Golgi Plug in 10 mL of cRPMI) and a 2X PMA-I cocktail (20 μL of 100x PMA, 20 μL of 1000x Ionomyocin, and 20 μL of 1000x Golgi Plug in 10 mL of cRPMI) were prepared. Following the resting incubation, 100 μL of stimulation cocktail was added to the appropriate cell samples and placed in a 37 °C incubator for 4 h.

### Live stain and fixation

Cells were transferred from the 96 well plate to a PCR plate (Thermo Fisher) and centrifuged at 400 x g for 5 min at room temperature, making sure to aspirate the resulting supernatant in between each transfer. Once all cells were transferred to the new PCR plate, cells were centrifuged and resuspended in FBS Stain Buffer (BD Biosciences). FC Block Cocktail (Human TruStain FcX Fc Receptor Blocking Solution [Biolegend] and FBS Stain Buffer) was added to each sample and incubated for 10 min at 21 °C. Following the blocking incubation, Live Stain Cocktail, composed of Live/Dead-Aqua (1:100, Biolegend), CD8-PerCP-Cy5.5 (1:20, Biolegend), and CD14-PE-Dazzle594 (1:20, Biolegend) in FBS Stain Buffer, was added to each well, and the plate was incubated for a subsequent 30 min at 21 °C. Samples were washed twice via centrifugation and resuspension in 150 μL of FBS Stain Buffer, and finally resuspended in 60 μL of FBS Stain Buffer. 16% PFA was added to each sample for a final concentration of 1.6% PFA and incubated for 10 min at 21 °C to fix the cells. After fixation, cells were washed as before and resuspended in FBS Stain Buffer in preparation for intracellular staining.

### Permeabilization and intracellular staining of PBMCs

Cold 1X Perm Buffer I (BD Biosciences) was added to each sample and mixed by pipetting. Samples were centrifuged, aspirated, and resuspended in 150 μL of cold Perm Buffer I before a 10-min incubation period at 4 °C. Incubated samples were centrifuged and resuspended in an intracellular staining cocktail composed of CD3-APC (1:400, Biolegend), CD4-PE (1:200, Biolegend), CD8-PerCP-Cy5.5 (5 μL, BD Biosciences), IFNγ-A488 (1:20, Biolegend), IL-4-BV421 (1:50, Biolegend), and IL-17-PE-Cy7 (1:50, Biolegend) diluted in Perm Buffer for 60 min at 4 °C. Following incubation, samples were washed twice using Perm Buffer I and once using FBS Stain Buffer (centrifugation at 400 x g for 5 min at 21 °C). After the final washing step, each sample was resuspended in cold FBS stain buffer and was characterized using flow cytometry.

### Flow Cytometry

Flow cytometry samples were acquired on a custom 4-laser BD LSRII flow cytometer equipped with the BD FACSDiva Software (BD Biosciences).

### Statistical analysis

Cellular yield was defined as the number of cells per mL of input blood both before and after cryopreservation while cellular recovery was the ratio of cells present after cryopreservation compared to prior. The cell viability was calculated as the percentage of living cells compared to the total cell count. Analysis of flow cytometry data for comparison of immune cell populations and cytokine productions were conducted using Cytobank.

Statistical comparisons between CPT and LP tubes were performed using R. The Wilcoxon Signed Rank Test was used to compare the means of repeated measurements on a single sample. Three-way ANOVA tests were used to identify any significant differences due to donor, operator, or method variability. For each parameter, a sum square was calculated for each independent variable (donor, method, and operator) to identify the deviation of each observation from the mean. The sum square contribution of each independent variable is calculated as sum square divided by the total sum square. From each sum square, a calculated *F* value was used to determine if a relationship exists between the dependent variables and the donor, method, and operator. A *p*-value is associated with the *F*-value, and any *p*-values less than 0.05 were considered to be statistically significant.

## Supplementary information

**Additional file 1: Figure S1.** Hematology analyzer data shows equivalent PBMC yield and post-thaw recovery using CPT or LP Tubes. **(A-C)** Three healthy donor blood draws of six tubes each were split between three operators for parallel PBMC isolation using CPT and Lymphoprep Tube methods. Total white blood cells in PBMCs were counted by a Sysmex XE-2100 automated hematology analyzer immediately after isolation (pre-freeze) and after cryopreservation and recovery (post-thaw). **(A)** The yield of PBMCs per mL of input blood pre-freeze. **(B)** The yield of PBMCs per mL of input blood post-thaw. **(C)** Percent of cell recovery post-thaw. Horizontal lines indicate mean +/− SEM. Donors 1,2, 3 are depicted by red, black and blue dots respectively. Three dots are shown per donor which represent single samples processed by three different operators. For statistical analysis of this data see Table S1.

**Additional file 2: Table S1.** Statistical analysis shows choice of CPT or LP method is not the major contributor to variance for yield, and recovery. Anova variance for contribution of donor, method (CPT or LP), and operator on cell yield pre-freeze, cell yield post-freeze, and recovery measured by Sysmex XE-2100 automated hematology analyzer. Sum squares, sum square contributions, *F* values, and *P* values are shown for each parameter measured (rows).

## Data Availability

The datasets used and/or analyzed during the current study are available from the corresponding author on reasonable request.

## Abbreviations

AO: Acridine orange
BD: Becton Dickinson
CEF: Cytomegalovirus, Epstein-Barr virus, and Influenza virus
CPT: Cell Preparation Tubes
cRPMI: Complete RPMI
DMSO: Dimethyl sulfoxide
FBS: Fetal bovine serum
LP: Lymphoprep
PBMC: Peripheral blood mononuclear cells
PBS: Phosphate-buffered saline
PFA: Paraformaldehyde
PI: Propidium Iodine
PMA-I: Phorbol-myristate-acetate and ionomycin

## References

[1] Posevitz-Fejfár A, Posevitz V, Gross CC, Bhatia U, Kurth F, Schütte V (2014). Effects of blood transportation on human peripheral mononuclear cell yield, phenotype and function: implications for immune cell biobanking. PLoS One.

[2] Kleeberger CA, Lyles RH, Margolick JB, Rinaldo CR, Phair JP, Giorgi JV (1999). Viability and recovery of peripheral blood mononuclear cells cryopreserved for up to 12 years in a multicenter study. Clin Diagn Lab Immunol.

[3] Costantini A, Mancini S, Giuliodoro S, Butini L, Regnery CM, Silvestri G (2003). Effects of cryopreservation on lymphocyte immunophenotype and function. J Immunol Methods.

[4] Hønge BL, Petersen MS, Olesen R, Møller BK, Erikstrup C (2017). Optimizing recovery of frozen human peripheral blood mononuclear cells for flow cytometry. PLoS One.

[5] Disis ML, Dela Rosa C, Goodell V, Kuan L-YY, Chang JCC, Kuus-Reichel K (2006). Maximizing the retention of antigen specific lymphocyte function after cryopreservation. J Immunol Methods.

[6] Longo DM, Louie B, Wang E, Pos Z, Marincola FM, Hawtin RE (2012). Inter-donor variation in cell subset specific immune signaling responses in healthy individuals. Am J Clin Exp Immunol.

[7] Kleiveland CR (2015). Peripheral blood mononuclear cells. The impact of food bioactives on health.

[8] Sathaliyawala T, Kubota M, Yudanin N, Turner D, Camp P, Thome JJC (2013). Distribution and compartmentalization of human circulating and tissue-resident memory T cell subsets. Immunity.

[9] Reimann KA, Chernoff M, Wilkening CL, Nickerson CE, Landay AL, Connick E (2000). Preservation of lymphocyte immunophenotype and proliferative responses in cryopreserved peripheral blood mononuclear cells from human immunodeficiency virus type 1-infected donors: implications for multicenter clinical trials. Clin Diagn Lab Immunol.

[10] Currier JR, Kuta EG, Turk E, Earhart LB, Loomis-Price L, Janetzki S (2002). A panel of MHC class I restricted viral peptides for use as a quality control for vaccine trial ELISPOT assays. J Immunol Methods.

[11] Godoy-Ramirez K, Franck K, Mahdavifar S, Andersson L, Gaines H (2004). Optimum culture conditions for specific and nonspecific activation of whole blood and PBMC for intracellular cytokine assessment by flow cytometry. J Immunol Methods.

[12] Lenarczyk A, Helsloot J, Farmer K, Peters L, Sturgess A, Kirkham B. Antigen-induced IL-17 response in the peripheral blood mononuclear cells (PBMC) of healthy controls. Clin Exp Immunol. 2000;122(1):41–8. 10.1046/j.1365-2249.2000.01328.x. PMC1905747 11012616

[13] Corkum CP, Ings DP, Burgess C, Karwowska S, Kroll W, Michalak TI (2015). Immune cell subsets and their gene expression profiles from human PBMC isolated by vacutainer cell preparation tube (CPT) and standard density gradient. BMC Immunol.

[14] Ruitenberg JJ, Mulder CB, Maino VC, Landay AL, Ghanekar SA (2006). VACUTAINER CPT And Ficoll density gradient separation perform equivalently in maintaining the quality and function of PBMC from HIV seropositive blood samples. BMC Immunol.

[15] Grievink HW, Luisman T, Kluft C, Moerland M, Malone KE (2016). Comparison of three tsolation techniques for human peripheral blood mononuclear cells: cell recovery and viability, population composition, and cell functionality. Biopreserv Biobank.

