# Supplementary figures and images for: Functional comparison of PBMCs isolated by Cell Preparation Tubes (CPT) vs. Lymphoprep Tubes
Source: BMC Immunol. 2020 Mar 30;21:15. doi: 10.1186/s12865-020-00345-0 (PMC7106580; doi:10.1186/s12865-020-00345-0)

Figure S1

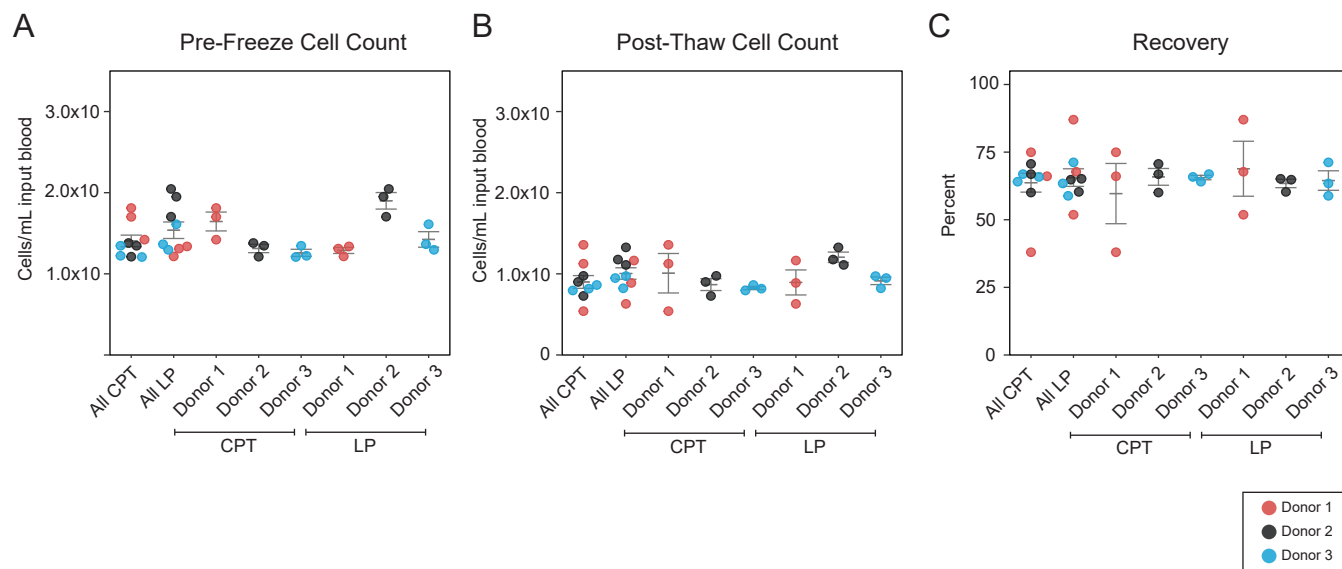

Supplement: Supplementary file 1 — Additional file 1: Figure S1. Hematology analyzer data shows equivalent PBMC yield and post-thaw recovery using CPT or LP Tubes. (A-C) Three healthy donor blood draws of six tubes each were split between three operators for parallel PBMC isolation using CPT and Lymphoprep Tube methods. Total white blood cells in PBMCs were counted by a Sysmex XE-2100 automated hematology analyzer immediately after isolation (pre-freeze) and after cryopreservation and recovery (post-thaw). (A) The yield of PBMCs per mL of input blood pre-freeze. (B) The yield of PBMCs per mL of input blood post-thaw. (C) Percent of cell recovery post-thaw. Horizontal lines indicate mean +/− SEM. Donors 1,2, 3 are depicted by red, black and blue dots respectively. Three dots are shown per donor which represent single samples processed by three different operators. For statistical analysis of this data see Table S1. [file 12865_2020_345_MOESM1_ESM.pdf]
